# Supplementary figures and images for: Visualisation tools for dependent peptide searches to support the exploration of in vitro protein modifications
Source: PLoS One. 2020 Jul 8;15(7):e0235263. doi: 10.1371/journal.pone.0235263 (PMC7343161; doi:10.1371/journal.pone.0235263)

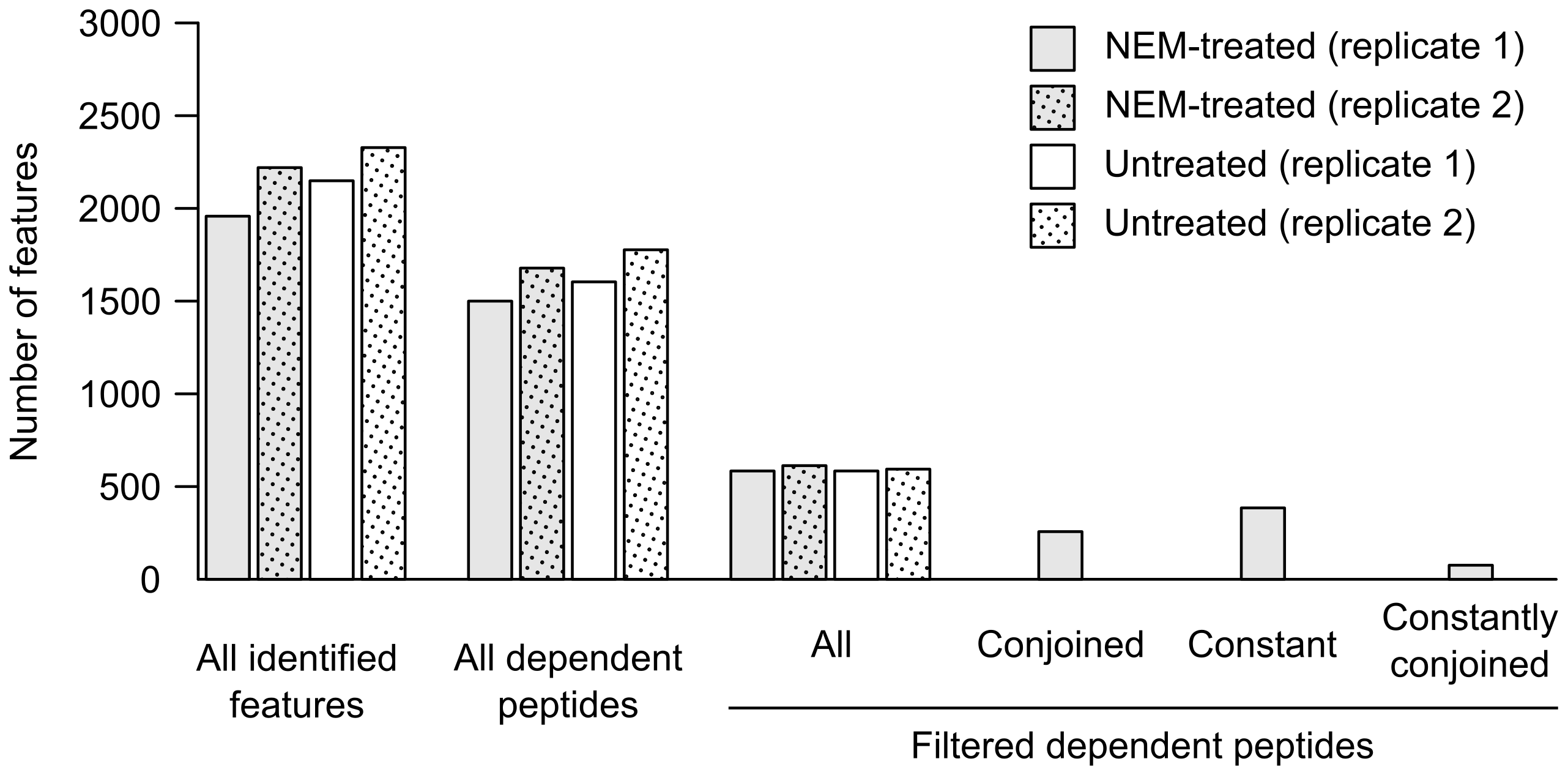

Supplement: S1 Fig — After filtering, the numbers of dependent peptides (DPs) were all similar (no two counts differed by more than 5%). ‘Conjoined’ DPs were those detected in analysis 1 of NEM-treated BSA and not detected in analysis 1 of untreated BSA. ‘Constant’ DPs were those detected in both analyses of NEM-treated BSA. ‘Constantly conjoined’ DPs were those detected in both analyses of NEM-treated BSA and not detected in either analysis of untreated BSA. (TIF) [file pone.0235263.s001.tif]

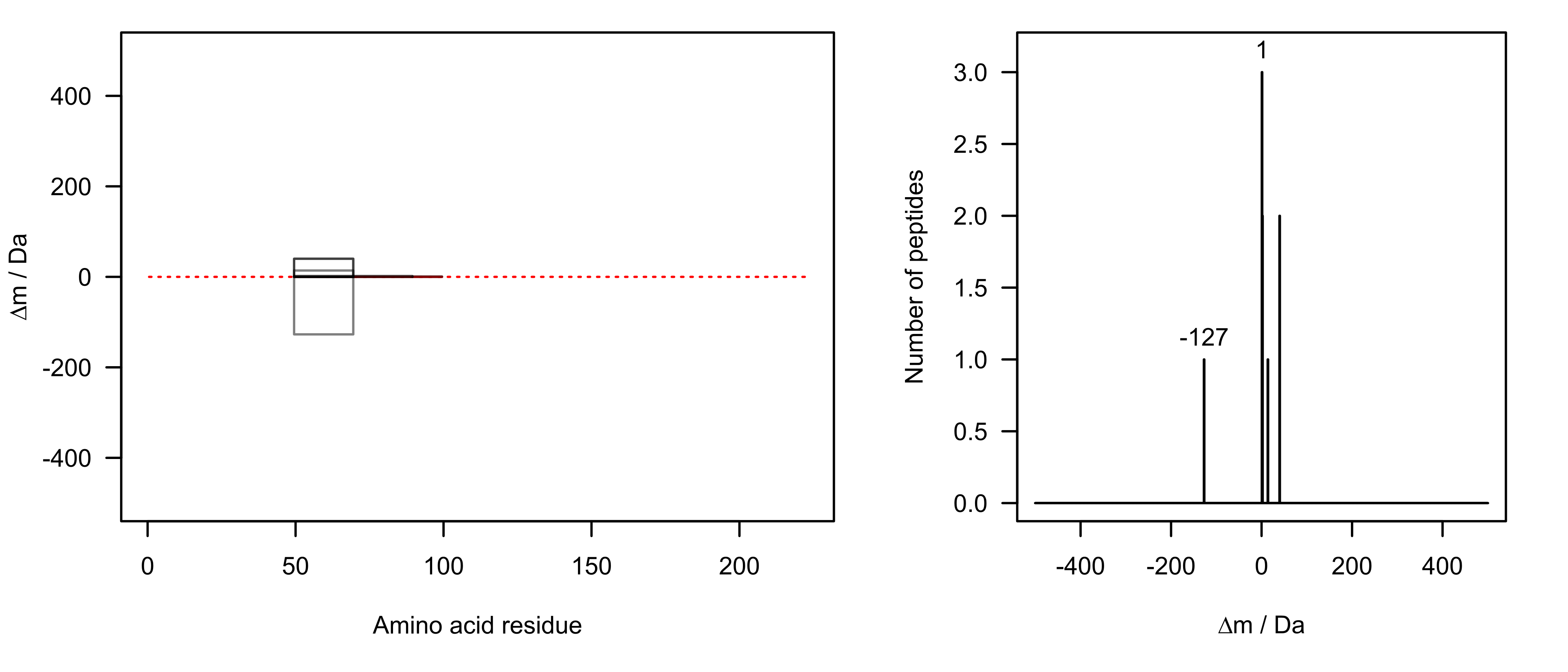

Supplement: S2 Fig — MaxQuant identified three putative deamidations and a putative methylation. Two of the deamidations were localised to asparagine residues. (TIF) [file pone.0235263.s002.tif]

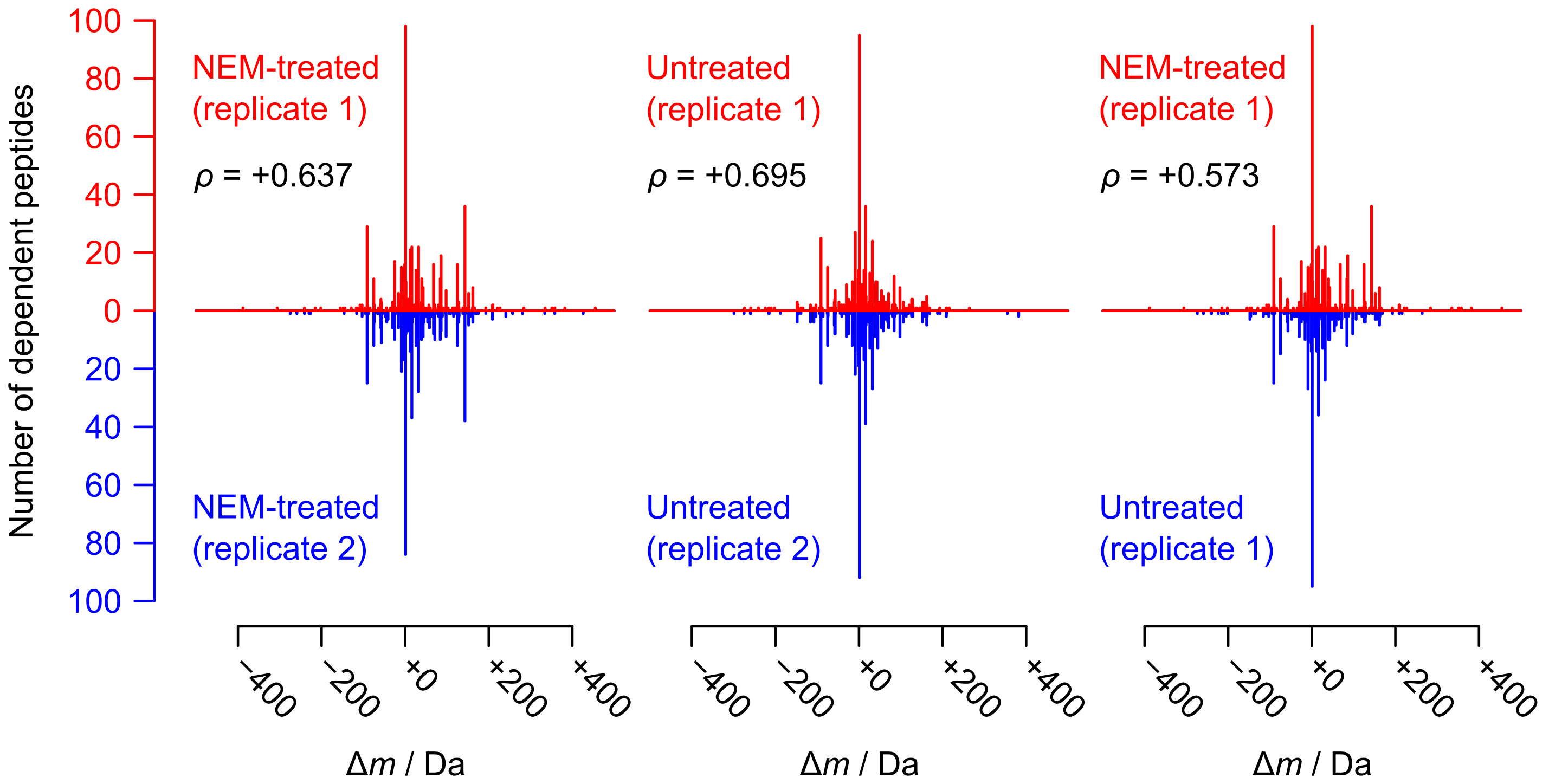

Supplement: S3 Fig — Three pairwise comparisons are shown: treated/treated, untreated/untreated and treated/untreated. The treated/untreated pair shown is the least similar of four possible combinations. ρ = Spearman correlation coefficient. (TIF) [file pone.0235263.s003.tif]

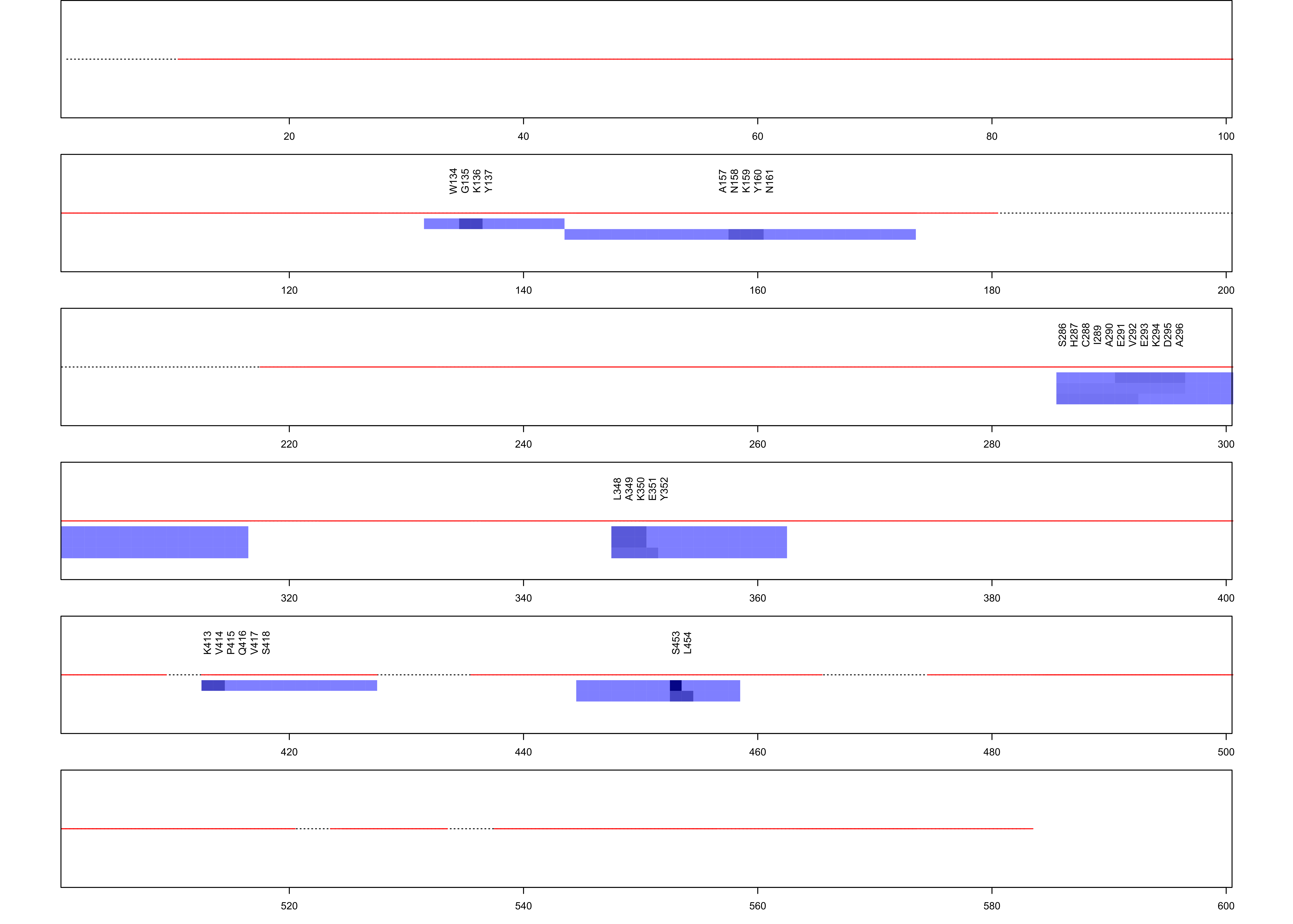

Supplement: S4 Fig — X-axis values refer to positions in the protein sequence. (TIF) [file pone.0235263.s004.tif]

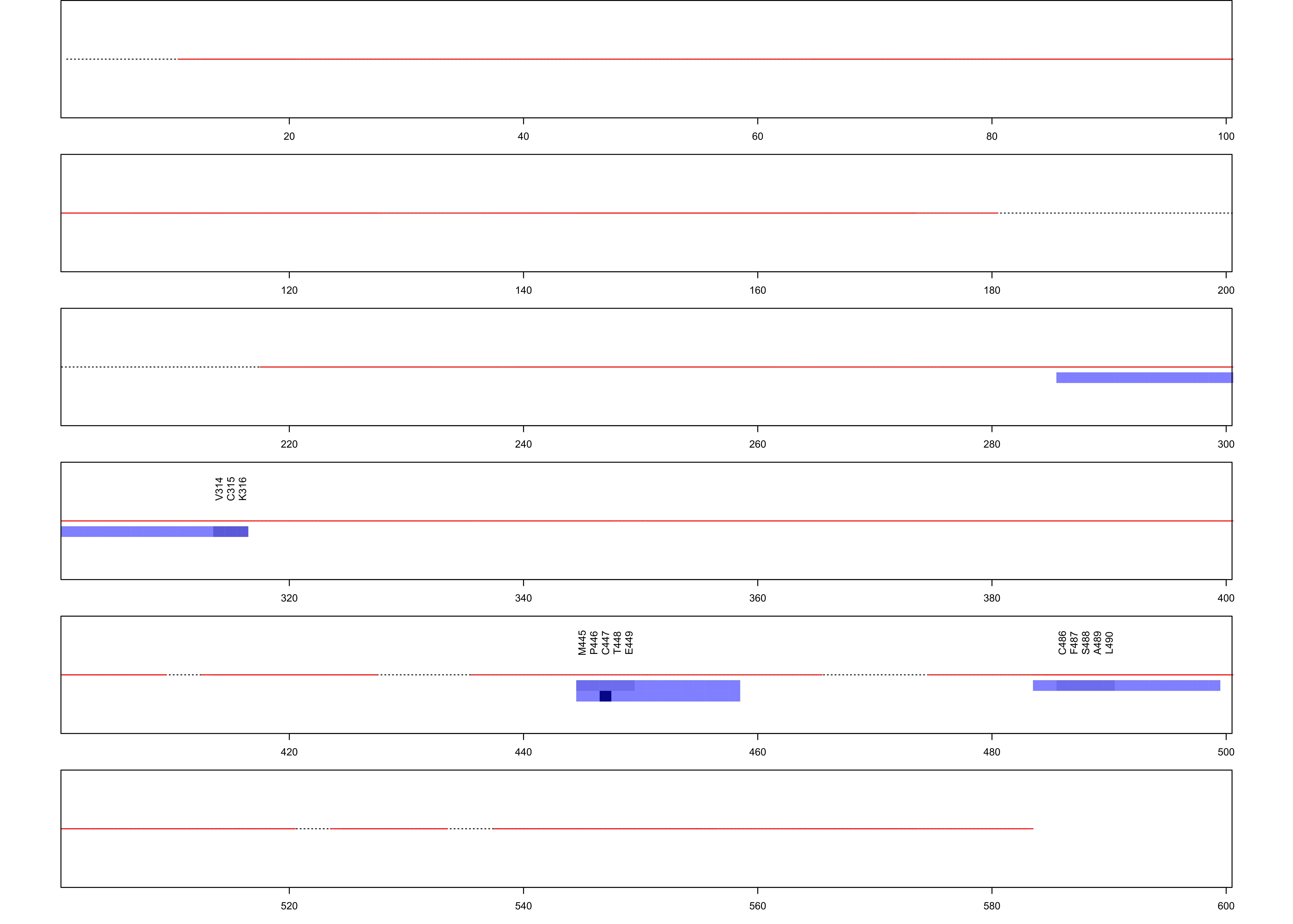

Supplement: S5 Fig — X-axis values refer to positions in the protein sequence. (TIF) [file pone.0235263.s005.tif]

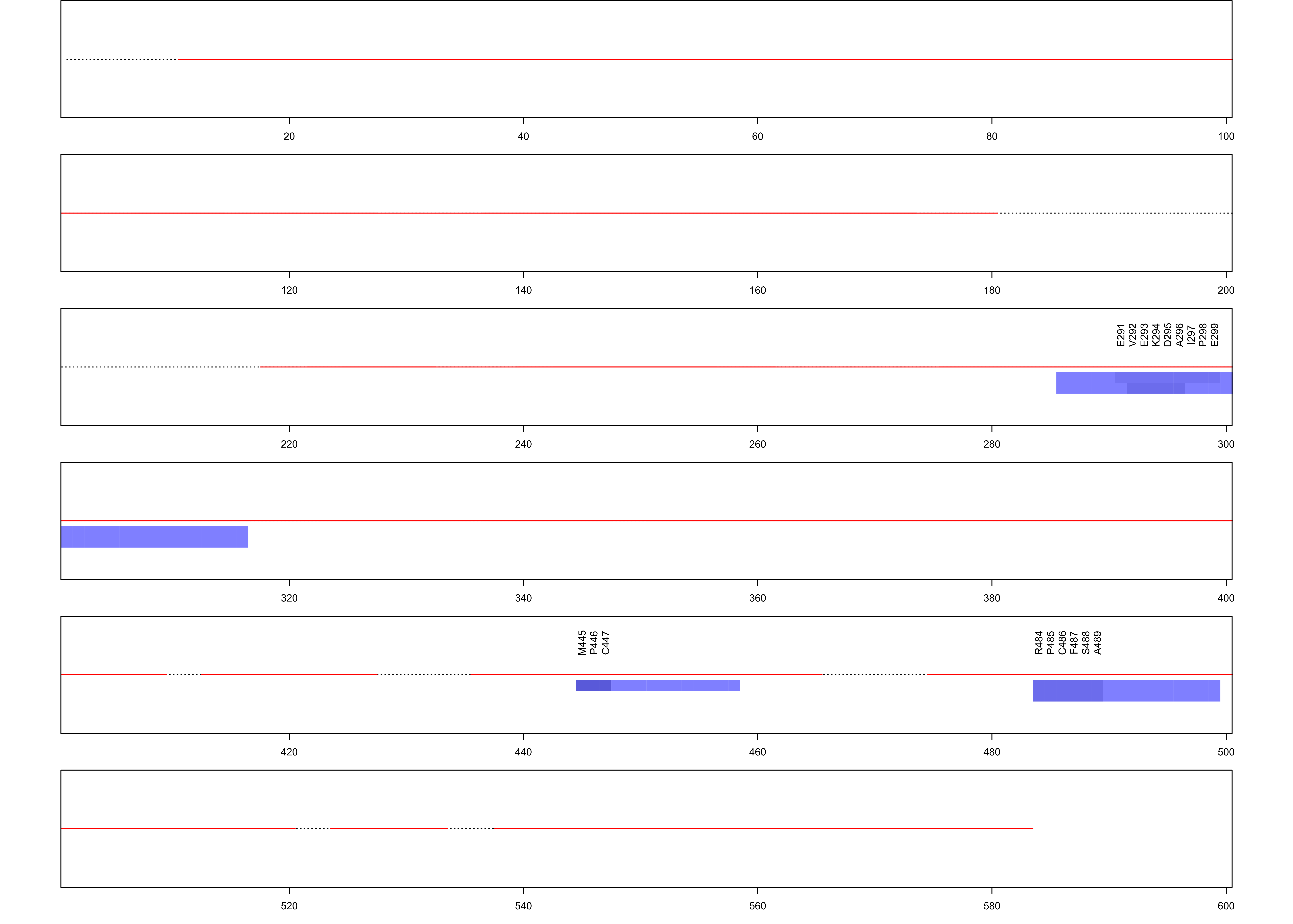

Supplement: S6 Fig — X-axis values refer to positions in the protein sequence. (TIF) [file pone.0235263.s006.tif]

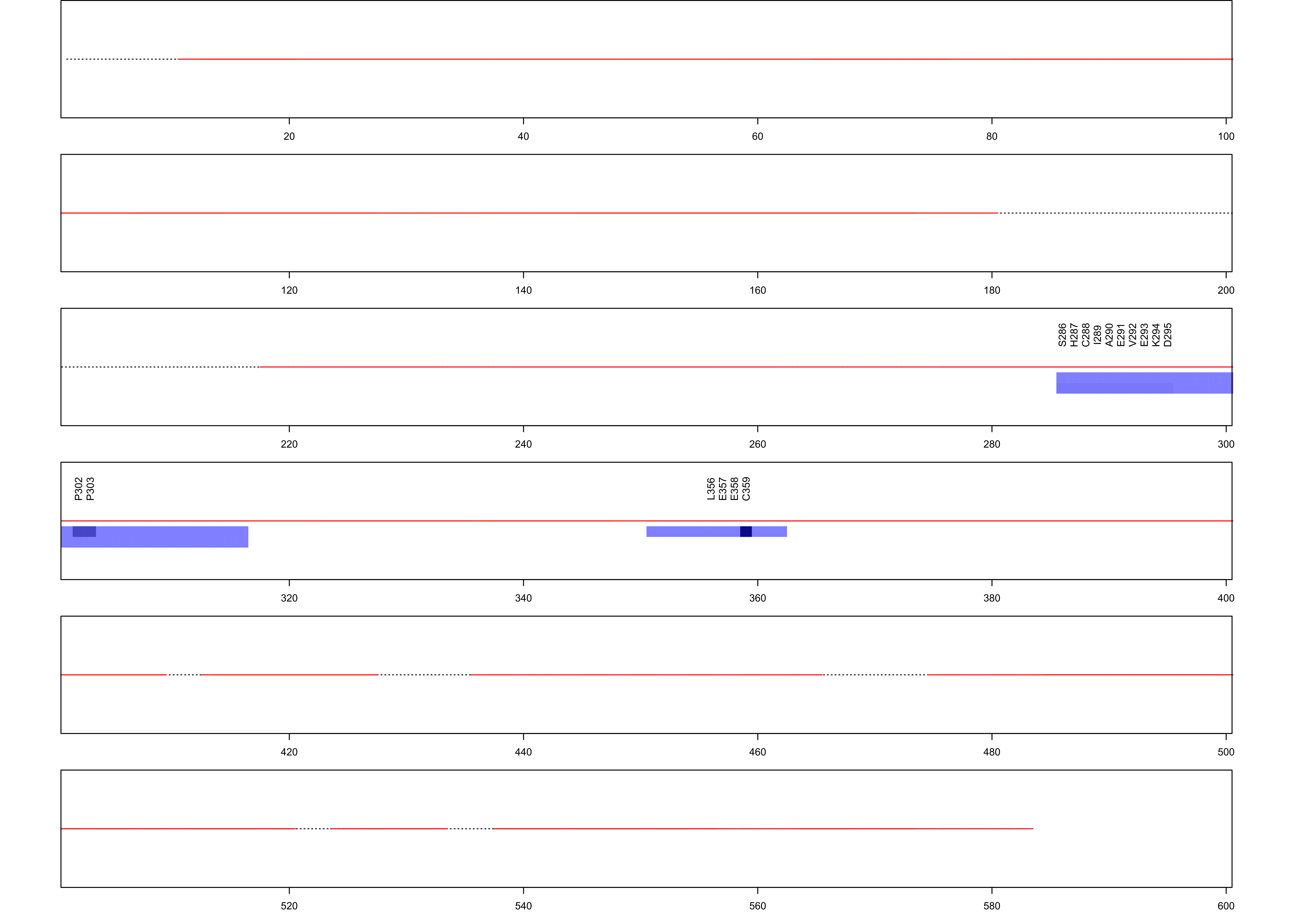

Supplement: S7 Fig — X-axis values refer to positions in the protein sequence. (TIF) [file pone.0235263.s007.tif]

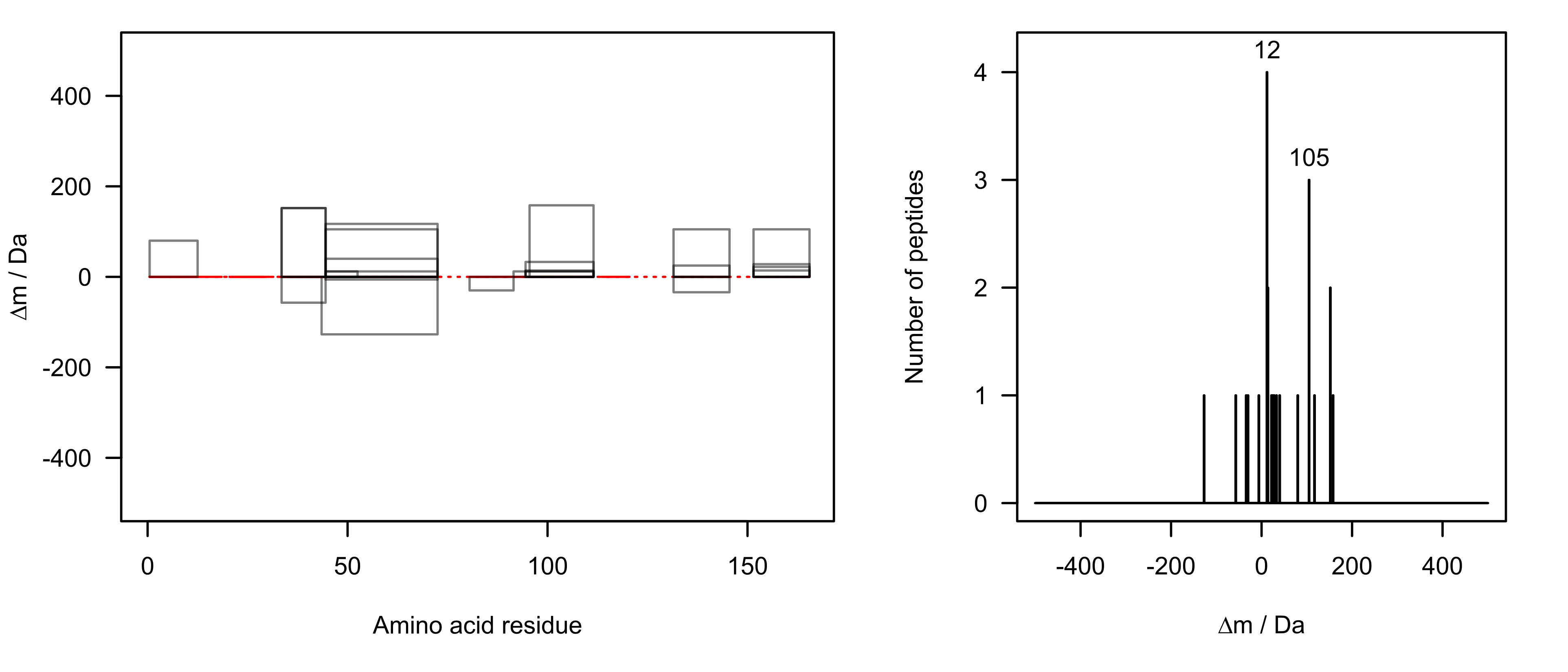

Supplement: S8 Fig — The nominal Δm of +105 Da is consistent with pyridylethylation of non-cysteine residues. (TIF) [file pone.0235263.s008.tif]

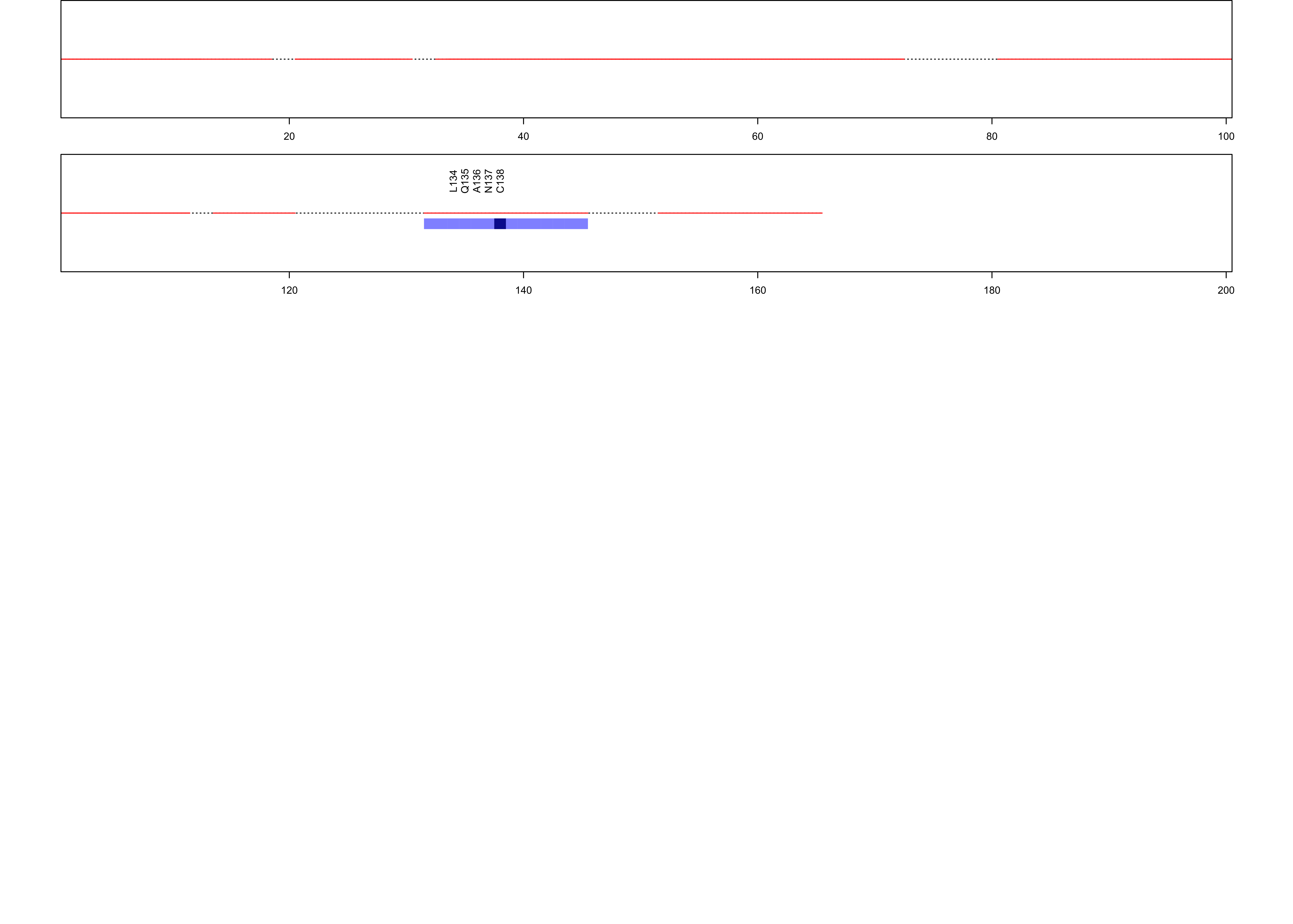

Supplement: S9 Fig — X-axis values refer to positions in the protein sequence. (TIF) [file pone.0235263.s009.tif]

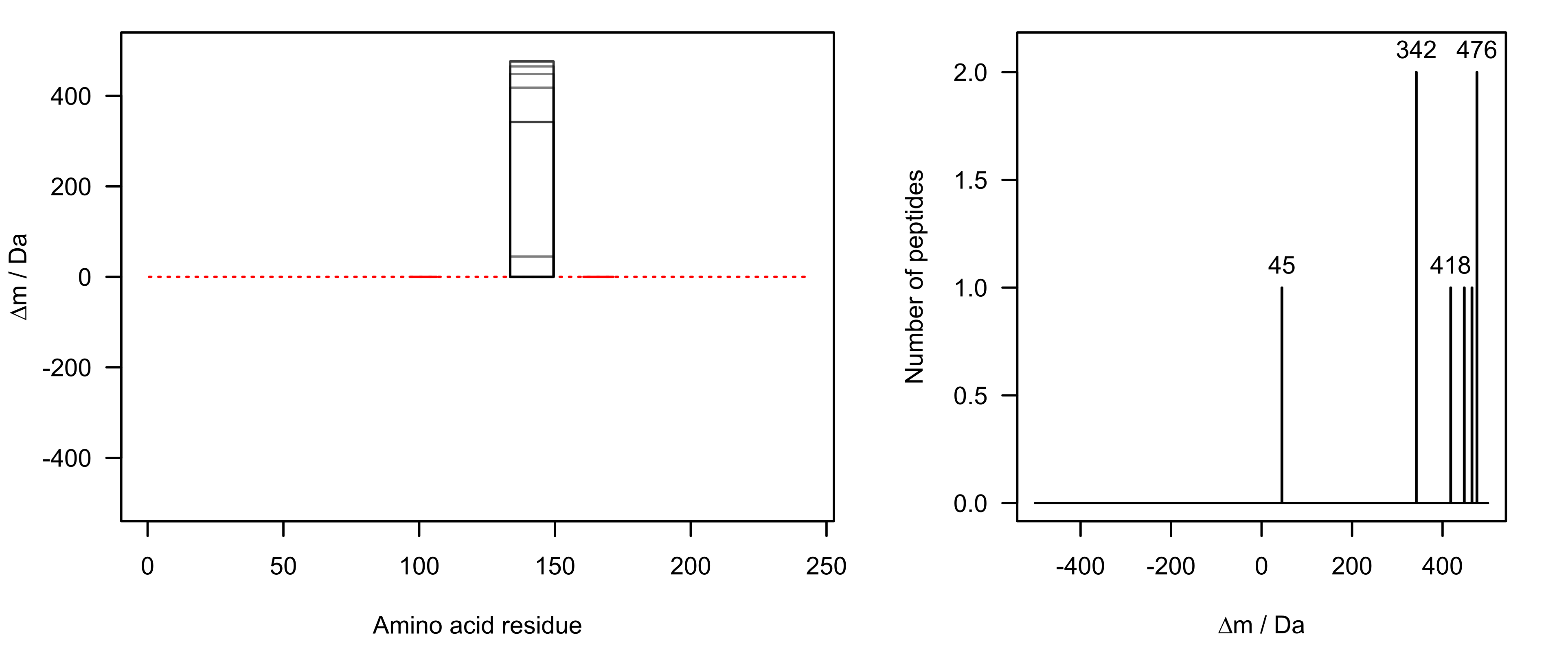

Supplement: S10 Fig — (TIF) [file pone.0235263.s010.tif]

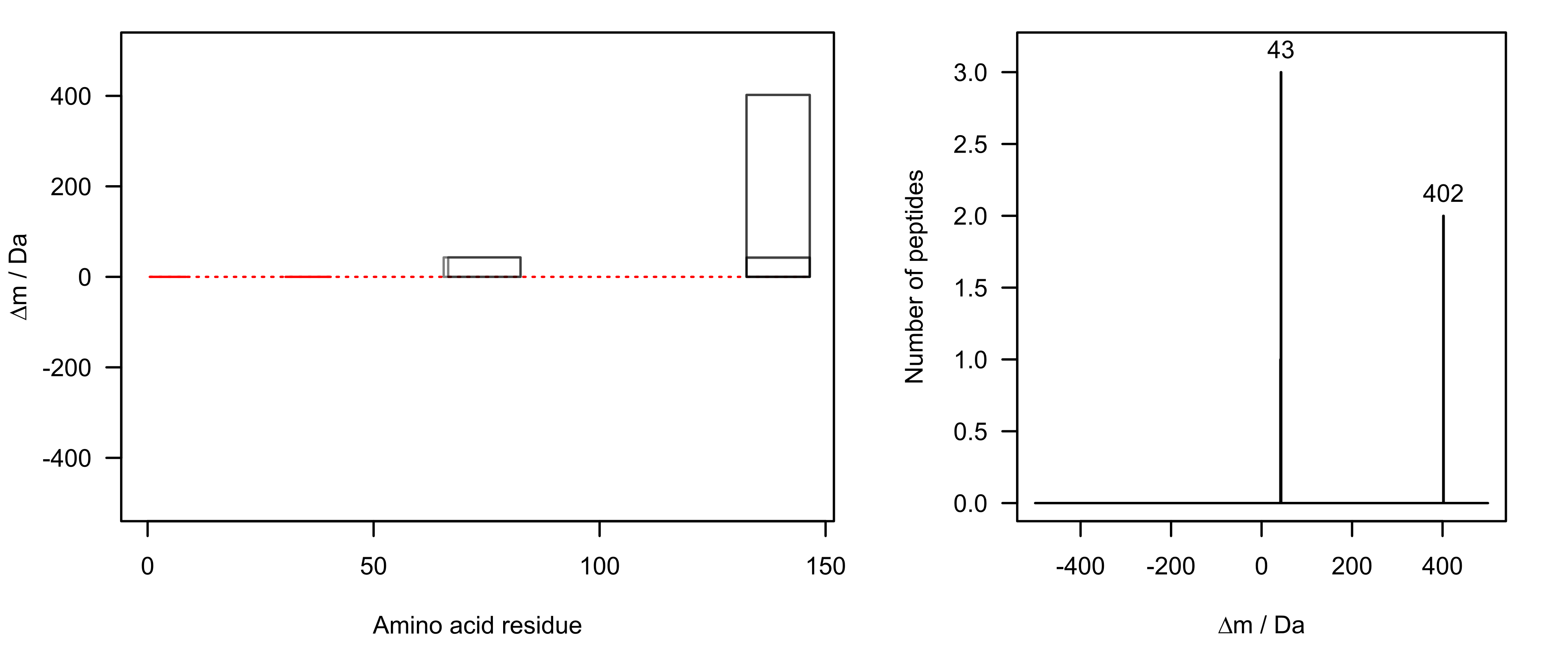

Supplement: S11 Fig — All six dependent peptides also mapped to the sequence of haemoglobin β-chain (Fig 3). (TIF) [file pone.0235263.s011.tif]

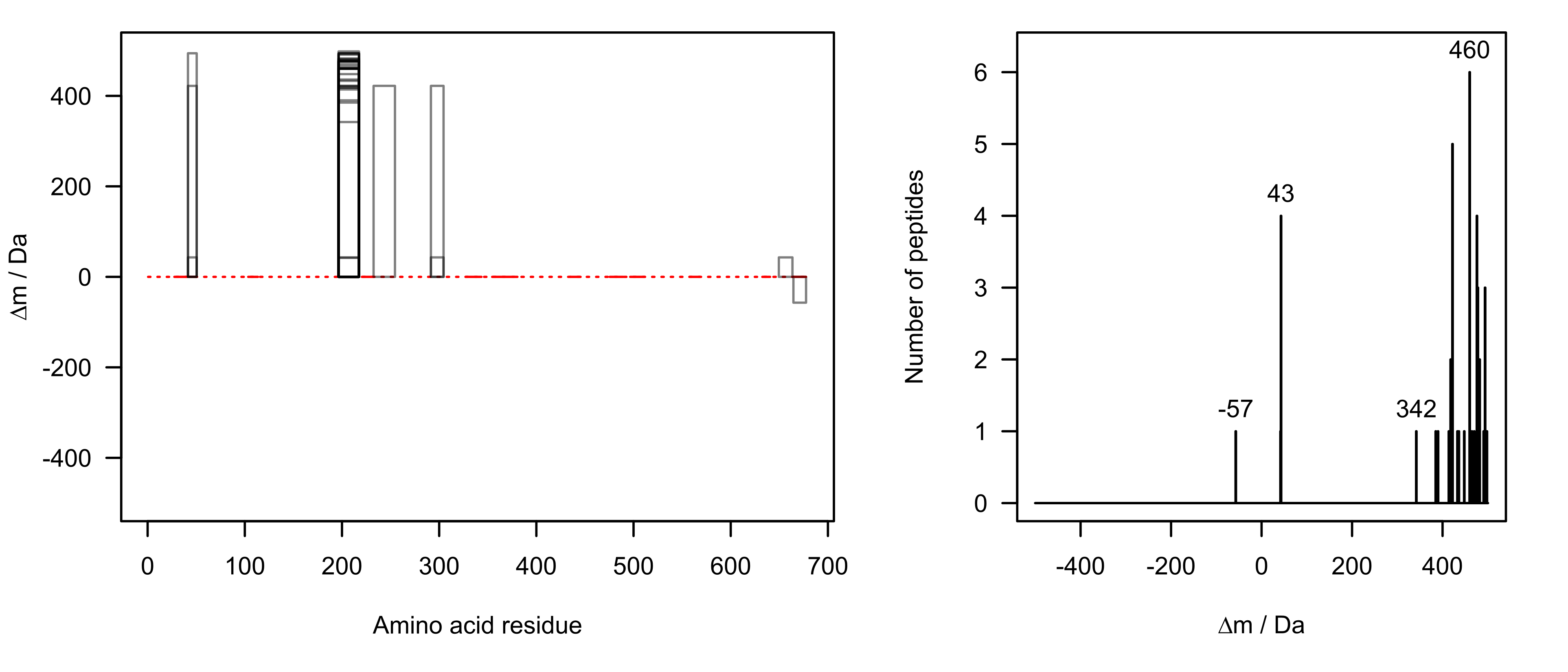

Supplement: S12 Fig — (TIF) [file pone.0235263.s012.tif]

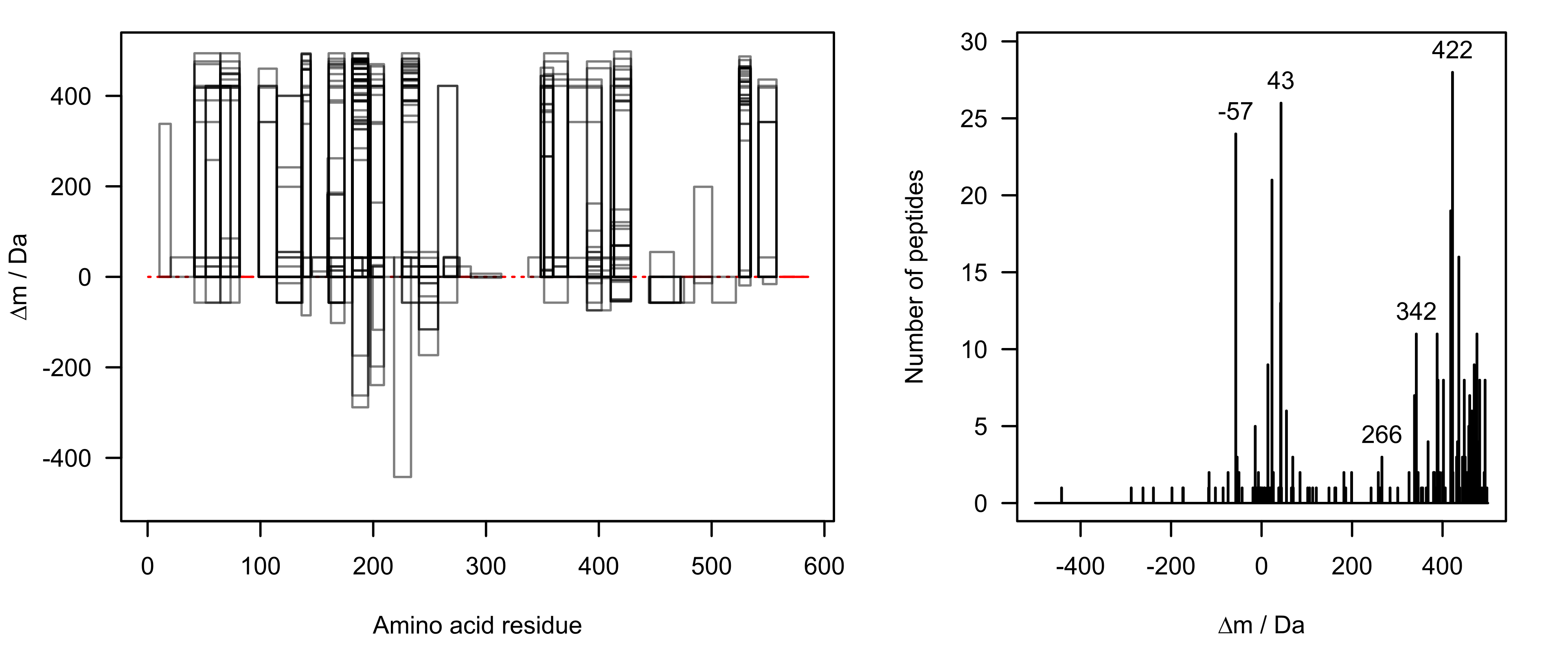

Supplement: S13 Fig — (TIF) [file pone.0235263.s013.tif]

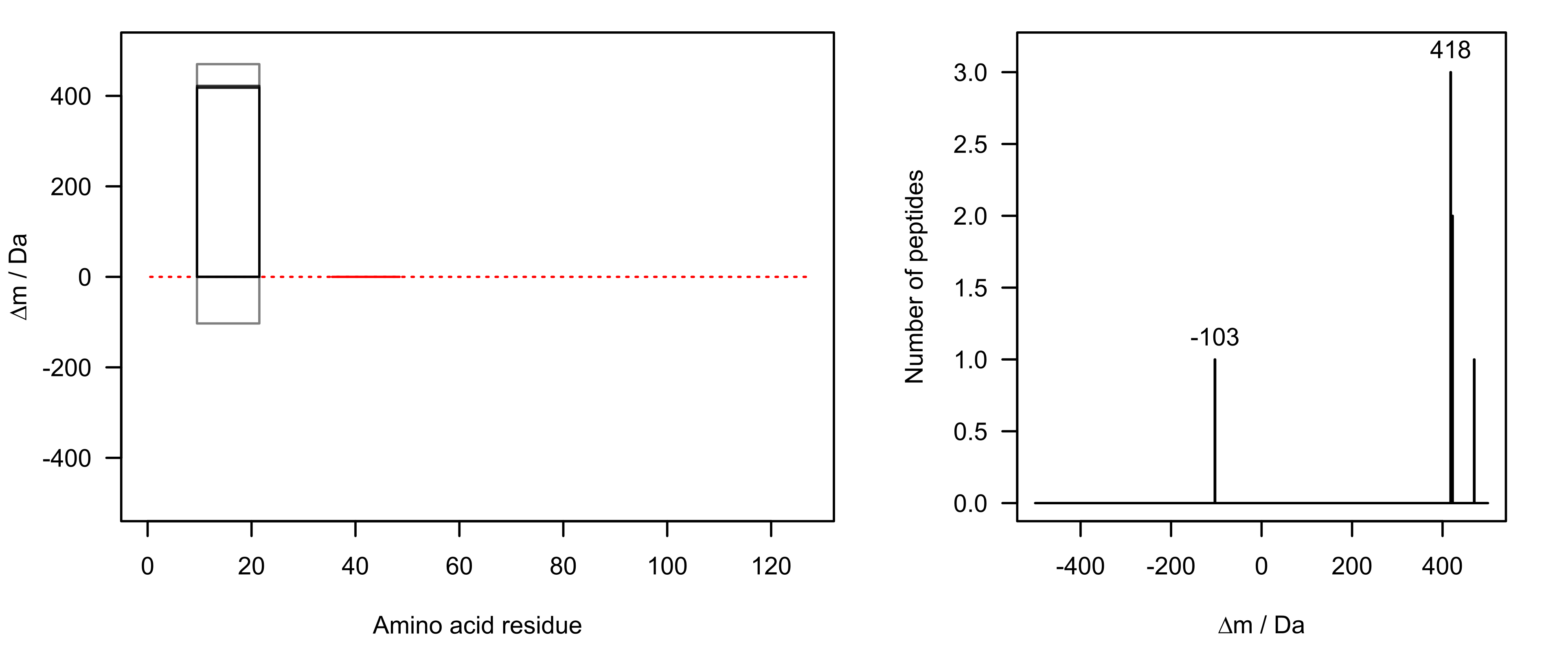

Supplement: S14 Fig — (TIF) [file pone.0235263.s014.tif]

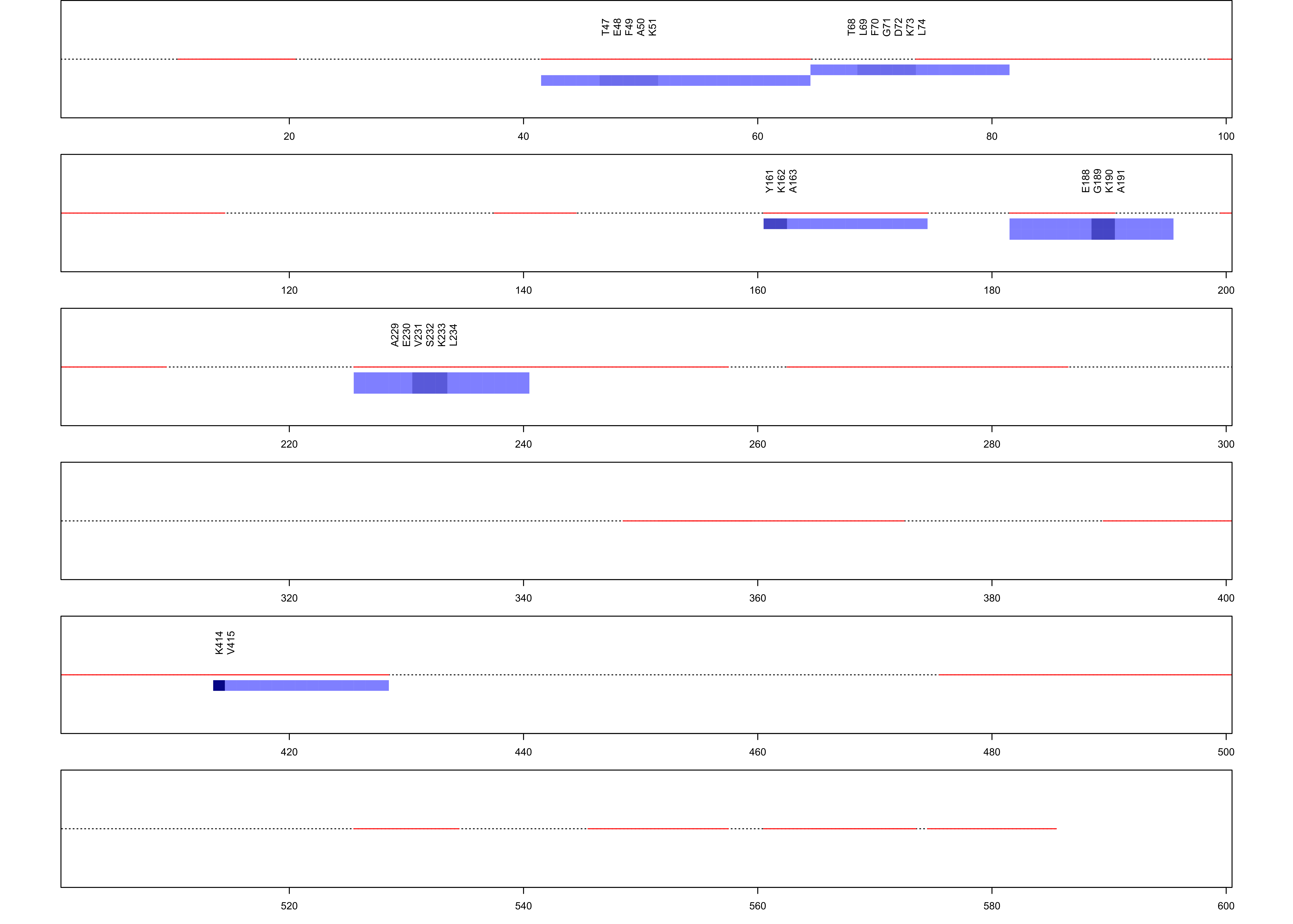

Supplement: S15 Fig — X-axis values refer to positions in the protein sequence. (TIF) [file pone.0235263.s015.tif]
