# Supplementary material for: Visualisation tools for dependent peptide searches to support the exploration of in vitro protein modifications
Source: PLoS One. 2020 Jul 8;15(7):e0235263. doi: 10.1371/journal.pone.0235263 (PMC7343161; doi:10.1371/journal.pone.0235263)
Supplement: S1 Text — Chemicals; Preparation of BSA adducts; Sample preparation for mass spectrometry; Nano liquid chromatography and mass spectrometry; Enumeration of tryptic peptides; Calculation of maximum peptide mass; Contaminant databases; Comparison of dependent-peptide and variable-modification search results; Figure preparation; References; Table A (Gradient elution timetable); Table B (Criteria used to filter dependent-peptide search results); Table C (Explanatory notes to accompany scripts). (PDF) [file pone.0235263.s023.pdf]

SUPPORTING INFORMATION

File S1 Text. Supplementary methods for 'Visualisation tools for dependent peptide searches to support the exploration of *in vitro* protein modifications'

G. W. Preston[1,2,#*], L. Yang[2], D. H. Phillips[1] and C. S. Maier[2*]

[1] MRC-PHE Centre for Environment & Health, Department of Analytical, Environmental & Forensic Sciences, School of Population Health & Environmental Sciences, Faculty of Life Sciences & Medicine, King's College London, London, United Kingdom

[2] Department of Chemistry, Oregon State University, Corvallis, OR, United States of America

[#] Current address: Stoller Biomarker Discovery Centre, University of Manchester, Manchester, United Kingdom

[*] Corresponding authors. E-mail: george.preston@kcl.ac.uk, claudia.maier@oregonstate.edu

Contents

Chemicals 2
Preparation of BSA adducts 2
Sample preparation for mass spectrometry 2
Nano liquid chromatography and mass spectrometry 3
Enumeration of tryptic peptides 4
Calculation of maximum peptide mass 4
Contaminant databases 4
Comparison of dependent-peptide and variable-modification search results 5
Figure preparation 5
References 11

Table A. Gradient elution timetable 4
Table B. Criteria used to filter dependent-peptide search results 7
Table C. Explanatory notes to accompany scripts 8

Chemicals

Bovine serum albumin (BSA) was purchased from ThermoFisher Scientific (Massachusetts, USA) as a 2 mg mL^{-1} aqueous solution (product number 23209) also containing 0.9% (*w*/*v*) sodium chloride and 0.05% (*w*/*v*) sodium azide [*caution: sodium azide is toxic and reactive*]. The solution was lyophilised and stored at −20 °C prior to use. UHPLC-grade solvents (acetonitrile and water) were also from ThermoFisher Scientific. *N*-Ethylmaleimide (NEM) was purchased from Alfa Aesar (Massachusetts, USA). Iodoacetamide was purchased from Bio-Rad (California, USA). Acetone (HPLC grade) was purchased from Merck (New Jersey, USA). Modified porcine trypsin (sequencing grade) was purchased from Promega (Wisconsin, USA). DL-1,4-Dithiothreitol (DTT) was purchased from Sigma-Aldrich (Missouri, USA). RapiGest SF surfactant was purchased from Waters (Massachusetts, USA). General laboratory reagents were purchased from reputable suppliers and were of sufficient purity for analytical work.

Preparation of BSA adducts

A solution of NEM (50 nmol) in potassium phosphate buffer (100 mM, pH 7.4) (25 µL) [*or phosphate buffer alone*] was added to a solution of BSA (50 µg) in the same buffer (25 µL). The BSA solution also contained sodium chloride (154 mM) and sodium azide (8 mM) (see above). The mixture was vortexed and left to stand at ambient temperature for 160 min. A solution of DTT (50 nmol) in the phosphate buffer (5 µL) [*or phosphate buffer alone*] was added to scavenge unreacted NEM, and the mixture was vortexed and left to stand at ambient temperature for 1 h. The reaction medium was exchanged for fresh phosphate buffer (Amicon Ultra-0.5 centrifugal filter units, 10 kDa cut-off; four tenfold concentration-dilution cycles). The phosphate buffer was then exchanged for a 50 mM aqueous solution of ammonium bicarbonate (three tenfold concentration-dilution cycles). The solution was filtered again to achieve a final protein concentration of 0.8 µg μL^{-1}.

Sample preparation for mass spectrometry

To 50 µL of filter retentate (40 µg of protein) was added a solution of DTT (280 nmol) in 50 mM aqueous ammonium bicarbonate (5 µL). The mixture was incubated at 56 °C for 1 h. After the mixture had cooled to ambient temperature, a solution of iodoacetamide (630 nmol) in 50 mM aqueous ammonium bicarbonate (5 µL) was added. The mixture was left to stand at ambient temperature, in the dark, for 1 h. Cold acetone (240 µL) was added, and the mixture was left to stand at −20 °C for 16 h. The resulting suspension was

centrifuged (8000 × *g*, 10 min, 4 °C) and the supernatant was removed. The pellet was allowed to air-dry for 10 min, before being dissolved in 39 µL of 50 mM aqueous ammonium bicarbonate containing 0.1% (*w*/*v*) RapiGest SF surfactant. A solution of trypsin (0.8 µg) in 50 mM aqueous ammonium bicarbonate (1 µL) was added, and the mixture was incubated at 37 °C for 20 h. Ten microlitres of a 5% (*v*/*v*) aqueous solution of trifluoroacetic acid were added to precipitate the surfactant, and the mixture was returned to the incubator for a further 45 min. The resulting suspension was centrifuged (15,000 × *g*, 7 min, 20 °C). A portion of the supernatant was diluted 40-fold with a 0.1% (*v*/*v*) aqueous solution of formic acid, and a portion of this diluted material was transferred to an autosampler vial in preparation for nano liquid chromatography and mass spectrometry.

Nano liquid chromatography and mass spectrometry

An Orbitrap Fusion Lumos mass spectrometer (ThermoFisher Scientific, Waltham, Massachusetts, USA) coupled with a nanoACQUITY liquid chromatograph (Waters, Milford, Massachusetts, USA) was applied to acquire all raw data. The eluents used for the liquid chromatography were 0.1% (*v*/*v*) aqueous formic acid (eluent A) and 0.1% (*v*/*v*) formic acid in acetonitrile (eluent B). Peptides (~20 ng) were loaded onto a Waters nanoACQUITY UPLC 2G C18 trap column (100 Å, 5 µm, 180 µm × 20 mm) in 3% (*v*/*v*) eluent B, at a flow rate of 5 µL min^{-1}, over a period of 5 min. Peptides were then separated on a Waters nanoACQUITY UPLC Peptide BEH C18 analytical column (130 Å, 1.7 µm, 100 µm × 100 mm) using gradient elution (see Table A). The mass spectrometer was operated under the positive ion polarity mode. The sample spray voltage and the ion transfer tube temperature were set at 2300 V and 300 °C, respectively. Mass spectra were acquired in the 'top speed' data-dependent mode. Master scans were done using the Orbitrap (resolution = 120,000, scan range = *m*/*z* 400-1500, automatic gain control target = 4×10^5) and data were acquired as the profile type. The most intense precursor ions of charge number two to seven were selected for fragmentation. Precursor ions were isolated in the quadrupole (isolation window = 1.6 *m*/*z* units) and fragmented by collision-induced dissociation (normalised collision energy = 35%). Fragment ions were analysed in the linear ion trap (automatic gain control target = 1×10^4) and data were acquired as the centroid type. Dynamic exclusion was used to exclude ions, including isotopologues, for which spectra had already been acquired ('if occurs within' = 30 s, exclusion duration = 60 s).

Table A. Gradient elution timetable

Time / min	Proportion (v/v) of eluent B in mobile phase (%)	Flow / nL min^{-1}
0	3	500
3	10	500
45	30	500
48	90	500
52	90	500
53	3	500
60	3	500

Enumeration of tryptic peptides

The sequence of mature BSA was digested *in silico* using PeptideMass [1]. The parameters were equivalent to those used for the corresponding Andromeda search. Methionine oxidation was added as a variable modification in R (version 3.6.0) [2]. The resulting table of peptides was filtered (length ≥ seven amino acid residues, molecular weight ≤ 4600 Da).

Calculation of maximum peptide mass

For each protein of interest, a table of theoretical peptides was prepared as described for BSA. Peptides of length ≥seven amino acid residues were ordered by mass, and the 95th percentile was computed. The maximum peptide mass was either the 95th percentile or Andromeda's default value (4600 Da), whichever was the greater.

Contaminant databases

Where necessary, MaxQuant's database of potential contaminants (contaminants.fasta) [3] was edited in R (version 3.4.0 or later) using the 'seqinR' package [4]. For the BSA study, all potential contaminants except BSA were included (244 proteins). For the rat cofilin-1 study, all potential contaminants were included (245 proteins). For the human plasma-protein study, 82 selected proteins were included (streptavidin, porcine trypsinogen; and 80 human proteins including keratins, dermokines, filaggrin and hornerin).

Comparison of dependent-peptide and variable-modification search results

A new modification, ‘Hydrolysed NESyl (CDEHKNQRSTY)’, was configured in Andromeda [3, 5]. The composition of the modification was ‘H(9) O(3) C(6) N’, the position was ‘anywhere’ and the type was ‘standard’. The specificities were amino acids with known or putative side-chain reactivity towards NEM (known according to Brewer and Riehm [6], or putative according to Figure 2). Glycine and amino acids with hydrocarbon side chains were excluded. The new modification was included as a variable modification in an Andromeda search. The other variable modifications were cysteine *S*-carbamidomethylation and methionine oxidation, and there were no fixed modifications. The *.raw file was from analysis 1 of NEM-treated BSA, and the database consisted of BSA and potential contaminants. The output table containing the search results [‘Hydrolysed NESyl (CDEHKNQRSTY)Sites.txt’] was filtered in R (Score > 80, PEP < 0.01, ‘Protein’ contains ‘4F5S’). Filtered results were matched to ‘constantly conjoined’ DPs by sequence and scan number (‘Best localization scan number’ = ‘DP Mod Scan Number’). Sites of modification within peptides were assigned manually on the basis of localisation probability. If multiple sites had the same probability, an assignment was made on the basis of chemical plausibility. For this purpose, the twenty common amino acids were grouped and prioritised as follows: (Cys, His or Lys [6]) > (Arg, Asn, Asp, Gln, Glu, Met, Ser, Thr, Trp or Tyr) > (Ala, Gly, Ile, Leu, Phe, Pro or Val). For the DPs only, possible sites of modification were further restricted: if $\Delta m = +86.04 \pm 0.01$ Da, site = Cys or not assigned; if $\Delta m = +143.06 \pm 0.01$ Da, site ≠ Cys (see ‘Results and discussion’ for the rationale).

Figure preparation

Figures S2 and S4-15. These figures are representative of the raw graphics generated by Scripts I-V. First, an SVG file was generated using an appropriate R script (see ‘Graphics devices’ in Table C). The file was opened in Inkscape (Free Software Foundation, version 0.91 or later) and a PNG image was exported. The PNG image was flattened in GNU Image Manipulation Program (GIMP Development Team, version 2.8.10) and exported in TIFF format.

Figure 2. This figure has been formatted for the journal pages. The following changes were made: panels were scaled and repositioned; margins were shrunk; the font size was increased; annotations were repositioned; lines were added to link annotations to data; borders were removed; line widths and styles were changed; tick-mark

lengths were changed; an x-axis title was added. All changes were made in Inkscape (version 0.91 or later). The final image was processed as described for Figures S2 and S4-15.

Figures 1, 3 and S3. These figures combine results for multiple data sets or modes of visualisation. Relevant raw graphics were combined in Inkscape. The following changes were made: panels were lettered (Figures 1 and 3); signs were added to annotations (Figures 1B, 1D, 1F and 3B); selected annotations were removed for clarity (Figures 1B, 1D, 1F and 3B); line colours were changed (Figure S3); histograms were rotated and labelled (Figure S3); other formatting was applied as described for Figure 2. Final images were processed as described for Figures S2 and S4-15.

Table B. Criteria used to filter dependent-peptide search results

Criterion	Details
‘DP Mass Difference’ is a numeric value	Isolates the DPs.
−500.5 Da ≤ ‘DP Mass Difference’ < +500.5 Da	Shapes the Δm distribution for compatibility with R function *hist* [2]. The interval includes all values of Δm that round to an integer in the range −500 to +500 (boundary values rounded to the more positive integer). These integer values will become the mid-points of the frequency histogram’s cells.
‘DP Score’ > 80	Limits the number of incorrect identifications. The ‘DP Score’ is a measure of how well the fragment-ion spectrum of a potential DP matches the corresponding theoretical spectrum [7]. The threshold score of 80 is from the method of Lassak *et al*. [8]
‘DP PEP’ < 0.01	Limits the number of incorrect identifications. The ‘DP PEP’ is assumed to be a posterior error probability akin to the one described by Tyanova *et al*. [3]
‘DP Proteins’ contains the identifier of the protein of interest (e.g., ‘4F5S’)	Excludes features attributed unambiguously to contaminants.
‘DP Decoy’ ≠ ‘+’	Excludes features identified as decoy DPs (artificial constructs with reversed sequences [7]).
‘DP Modification’ ≠ ‘Carbamidomethyl’	Excludes DPs with the ‘Carbamidomethyl’ modification, on the basis that carbamidomethylation probably occurred during sample preparation.
If ‘DP AA’ = ‘C’, ‘DP Modification’ ≠ ‘CarbamidomethylDTT’	Excludes DPs with the putative ‘CarbamidomethylDTT’ modification [9], but only if this modification has been localised to a cysteine residue.
‘DP Modification’ does not contain ‘Cation’	Excludes metal cation adducts, on the basis that they were probably formed during sample preparation or analysis.
‘DP Modification’ ≠ ‘Loss of ammonia’	Excludes DPs with the modification ‘Loss of ammonia’, on the basis that ammonia was probably lost during mass spectrometry.
‘DP Modification’ ≠ ‘Loss of water’	Excludes DPs with the modification ‘Loss of water’, on the basis that water was probably lost during mass spectrometry.
‘DP Modification’ ≠ ‘Unmodified’	Excludes unmodified peptides.
‘DP Peptide Length Difference’ ≥ 0	Excludes truncated peptides.

Table C. Explanatory notes to accompany scripts*

Topic	Scripts	Details
**protein.ID**	All	The **protein.ID** is an identifier used to extract records (i.e., complete rows) from **all.peptides** or a variant thereof. In the present study, the **protein.ID** was either a Protein Data Bank identifier or a UniProtKB accession number.
allPeptides.txt files	All	allPeptides.txt files are loaded using *read.table*. Alternatively, they could be converted to *.csv files and loaded using *read.csv*. If there are two or more files, the corresponding objects are distinguished by prefixes ('treated_', 'control_', 'treated_replicate1_', etc.)
Graphics devices	All	The default graphics device is *windows* (displays graphics on-screen). According to the documentation [2], this device is Windows-specific. For figure preparation, we used *svg* (generates *.svg files).
The sliding window method	All	The 'sliding window', is driven by a nested *for*-loop. Each 'slide' reveals a new **segment** of the **protein.sequence**. The outer loop adjusts the window to fit the sequence of the *i*th peptide. The inner loop slides the window along the **protein.sequence**, one amino acid residue at a time. The *i*th window reveals a total of $p - (w_i - 1)$ segments, where p and w_i are the lengths of the protein and the window, respectively. If the *j*th 'view' through the *i*th window (i.e., the **segment**) matches the *i*th peptide, then further commands are triggered (see below).
Sequence coverage	All	A non-redundant list of observed peptide sequences is compiled from **all.peptides** or a variant thereof. Wherever possible, sequences from analyses of *untreated* protein are used, because these should provide the best sequence coverage (chemical treatments have the potential to 'delete' segments of the sequence). If two analyses of untreated protein are available, then only sequences that appear in both analyses are mapped. Solid 'peptides' (line segments) are mapped onto a dashed 'backbone'.
Mapping of DPs' Δm values to protein segments	I, II, III	Each dependent peptide is mapped onto the backbone as a rectangle with a partially-transparent border. The sliding window passes a set of coordinates to a matrix (= **vertices**), which is used by *polygon* to draw the rectangle. The *x*-coordinates are backbone loci. The *y*-coordinates are 0, 0, Δm and Δm.
Axis limits	All	The *x*-axis of the localisation plot auto-adjusts to accommodate the **protein.sequence**. The *y*-axis of the localisation plot has fixed limits (**delta.mass.axis.limits**), and the *x*-axis of the frequency histogram has these same fixed limits. The *y*-axis of the frequency histogram auto-adjusts to accommodate the tallest cell, plus an annotation.

Table continues on next page

* Names of R objects are in boldface. Names of functions are italicised.

Table C (cont.)

Topic	Scripts	Details
Auto-annotation of histograms	I, II, III	For the purpose of annotation, the **histogram** is divided up into ‘slices’ (groups of contiguous cells). **histogram.slices** is a table specifying how the histogram should be divided up, and how many (= *N*) annotations should made for each slice. A *for*-loop excises the *counts* from the *i*th slice of **histogram** (= **slice_counts**) and identifies the *N* tallest cells (= **cells.to.annotate**). Nominal Δm values are printed above these cells using *text*.
Pairwise comparisons of Δm values	II, III, IV	The **tolerance** (*t*) determines how close a pair of Δm values must be in order to be considered a match. An observed value (Δm_{obs}) is considered as matching an expected value (Δm_{exp}) if $\Delta m_{exp} - t < \Delta m_{obs} < \Delta m_{exp} + t$. Two observed Δm values (Δm_1 and Δm_2) are considered to match if they differ by less than 2*t* (see below). In the present study, we used $t = 0.01$ Da.<br>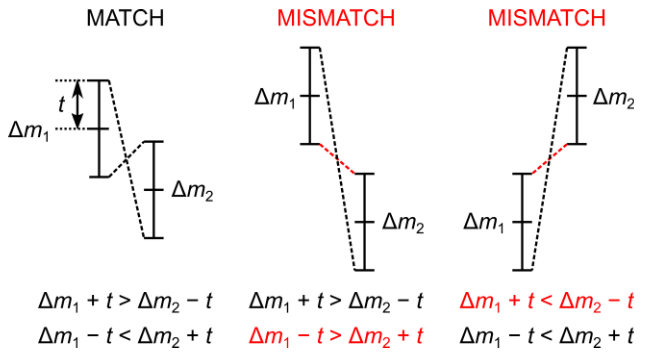
Subtracting ‘constant’ DPs	II	‘Constant’ DPs are identified *via* a round of DP-DP comparisons. Every DP in **treated_dependent.peptides** is compared to every DP in **control_dependent.peptides**. If the DPs match (same sequence AND $\Delta m_1 \approx \Delta m_2$), the corresponding row of **treated_dependent.peptides** is removed.
Identifying ‘constantly conjoined’ DPs	III, IV	‘Constantly conjoined’ DPs are identified *via* three rounds of DP-DP comparisons. In the first round, DPs from the *first analysis of treated protein* are compared to DPs from the *second analysis of treated protein*. When a match is observed, details of both DPs (sequence, Δm values, retention times) are copied to **treated_DP.constancy.results**, and eventually passed to **treated_constant.DPs**. By convention, DPs from the second analysis are matched to DPs from the first. This means that a single DP from the second analysis can end up being matched to *multiple* DPs from the first analysis. In such cases, the DP that is recorded in **treated_constant.DPs** will be the last one to have been matched.<br><br>In the second round, DPs that were found in *both analyses of treated protein* (as characterised by sequence and mean $\Delta m^{\dagger}$) are compared to DPs from the *first analysis of untreated protein*. In the third round, DPs that were found in *both analyses of treated protein* are compared to DPs from the *second analysis of untreated protein*. Any matches identified in the second and third rounds are recorded in **treated_constant.DPs**, and the corresponding rows of this data frame are removed.

Table continues on next page

† mean $\Delta m = (\Delta m_1 + \Delta m_2) / 2$

Table C (cont.)

Topic	Scripts	Details
Parsing 'DP Probabilities'	IV, V	Localisation probabilities are obtained by parsing a string called 'DP Probabilities'. The probabilities are excised and linked back to their corresponding protein sequence loci (i.e., numbered amino acid residues). Each string is split at the open-parenthesis symbols using *strsplit*, thereby generating the **primary.fragments**. Fragments containing close-parenthesis symbols are split again to generate **secondary.fragments**. Relevant fragments are then arranged in a table (= **peptide.probabilities**), and the peptide sequence loci are determined. Peptide sequence loci are converted to protein sequence loci using information passed from the sliding window.
Mapping of DPs and their localisation probabilities	IV, V	The mapping of a DP to a protein segment is triggered by a sliding window. For the *i*th DP, 'DP Probabilities' is parsed and a **peptide.probabilities** table is generated (see above). By convention, 'DP Probabilities' is for the feature detected in the *first analysis of treated protein*. Each DP is allocated a strip of elements in a matrix (elements = amino acid residues, row numbers = sequence loci), and then 'etched' into a blank copy of the matrix (= **transparency**) as a strip of ones. The localisation probabilities from **peptide.probabilities** are deposited in a corresponding zero matrix (= **protein.probabilities**). DPs with overlapping or contiguous sequences are allocated strips in different columns (max. five). To ensure that each DP is allocated its own discrete strip, elements *within* or *adjacent to* already-allocated strips are 'reserved' in a separate matrix (= **reservations**). The script keeps attempting to allocate a strip until it finds one that is fully 'unreserved'.<br><br>An *image* is prepared from **protein.probabilities**. A second *image*, prepared from **transparency**, is overlaid onto the first. In the resulting composite image, potential sites of modification (grey squares) will be visible within each DP (partially transparent strip). As with the other scripts, solid 'peptides' (line segments) are mapped onto a dashed 'backbone'. All potential sites of modification are annotated (one-letter amino acid symbol and number).<br><br>The image will normally need to be displayed across multiple panels. The number of panels (= **panel.count**) is calculated based on the number of characters in the **protein.sequence** (100 amino acid residues per panel). The different panels are effectively different views of the same image.
Script flexibility/ limitations	I, II, III	In theory, there is no limit to the number of DPs that Scripts I, II and III can visualise. In practice, the highest number for which visualisation was attempted was 584. In this case, visualisation of the 584th DP was confirmed.
	IV, V	Scripts IV and V arrange DPs in rows, up to a maximum of five rows. Overlapping or contiguous DPs are placed on different rows, which can lead to rows being skipped. Consequently, the number of available rows in a given region of the plot may be fewer than five. If any DPs appear on the bottom row, we would advise checking the relevant data frame (**treated_constantly.conjoined.DPs** or **dependent.peptides**) to make sure that all DPs have been visualised.

References

1. Wilkins MR, Lindskog I, Gasteiger E, Bairoch A, Sanchez J-C, Hochstrasser DF, et al. Detailed peptide characterization using PEPTIDEMASS – a World-Wide-Web-accessible tool. Electrophoresis. 1997;18(3-4):403-8. doi: 10.1002/elps.1150180314.

2. R Core Team. A language and environment for statistical computing. Vienna, Austria: R Foundation for Statistical Computing; 2017.

3. Tyanova S, Temu T, Cox J. The MaxQuant computational platform for mass spectrometry-based shotgun proteomics. Nat Protoc. 2016;11:2301-19. doi: 10.1038/nprot.2016.136.

4. Charif D, Lobry JR. SeqinR 1.0-2: A Contributed Package to the R Project for Statistical Computing Devoted to Biological Sequences Retrieval. In: Bastolla U, Porto M, Roman E, Vendruscolo M, editors. Structural Approaches to Sequence Evolution: Molecules, Networks, Populations. Biological and Medical Physics, Biomedical Engineering. New York: Springer Verlag; 2007. p. 207-32.

5. Cox J, Neuhauser N, Michalski A, Scheltema RA, Olsen JV, Mann M. Andromeda: A Peptide Search Engine Integrated into the MaxQuant Environment. J Proteome Res. 2011;10(4):1794-805. doi: 10.1021/pr101065j.

6. Brewer CF, Riehm JP. Evidence for possible nonspecific reactions between N-ethylmaleimide and proteins. Anal Biochem. 1967;18(2):248-55. doi: 10.1016/0003-2697(67)90007-3.

7. Mordret E, Dahan O, Asraf O, Rak R, Yehonadav A, Barnabas GD, et al. Systematic Detection of Amino Acid Substitutions in Proteomes Reveals Mechanistic Basis of Ribosome Errors and Selection for Translation Fidelity. Mol Cell. 2019;75(3):427-41. doi: 10.1016/j.molcel.2019.06.041.

8. Lassak J, Keilhauer EC, Fürst M, Wuichet K, Gödeke J, Starosta AL, et al. Arginine-rhamnosylation as new strategy to activate translation elongation factor P. Nat Chem Biol. 2015;11:266-70. doi: 10.1038/nchembio.1751.

9. Chalkley RJ, Baker PR, Medzihradszky KF, Lynn AJ, Burlingame AL. In-depth Analysis of Tandem Mass Spectrometry Data from Disparate Instrument Types. Mol Cell Proteomics. 2008;7(12):2386-98. doi: 10.1074/mcp.M800021-MCP200.
